# A 33.2 W High Beam Quality Chirped-Pulse Amplification-Based Femtosecond Laser for Industrial Processing

**DOI:** 10.3390/ma13122841

**Published:** 2020-06-24

**Authors:** Zhenao Bai, Zhenxu Bai, Xiaolong Sun, Yong Liang, Kun Wang, Duo Jin, Zhongwei Fan

**Affiliations:** 1 Aerospace Information Research Institute, Chinese Academy of Sciences, Beijing 100094, China; baizhenao@hotmail.com; 2 Beijing GK Laser Technology Co., Ltd., Beijing 102206, China; 3 National Engineering Research Center for DPSSL, Beijing 100094, China; 4 Hangzhou Yacto Technology Ltd., Hangzhou 311300, China; xlsun@yacto-tech.com (X.S.); yliang@yacto-tech.com (Y.L.); 5 Center for Advanced Laser Technology, Hebei University of Technology, Tianjin 300401, China; zxbai@hebut.edu.cn (Z.B.); jin_duo@outlook.com (D.J.); 6 MQ Photonics Research Centre, Department of Physics and Astronomy, Macquarie University, Sydney, NSW 2109, Australia; 7 School of Energy and Environmental Engineering, Hebei University of Technology, Tianjin 300401, China; wangkun@hebut.edu.cn; 8 University of Chinese Academy of Sciences, Beijing 100049, China

**Keywords:** femtosecond, photonic crystal fiber, chirped pulse amplification, laser processing

## Abstract

A photonic crystal fiber-based chirped pulse amplification delivering 272 fs pulses of 66.4 µJ energy at a repetition rate of 500 kHz is presented, resulting in an average/peak power of 33.2 W/244 MW. A single grating is adopted for the pulse width stretching and compression, which leads to high-compactness and low cost of the system. The output beam is near-diffraction-limited (*M*^2^ = 1.1 ± 0.05) with a power stability better than 0.5%. The cutting of alumina ceramic substrate and flexible printed circuit are demonstrated by using the laser system. The results indicate that the laser is competent for industrial applications.

## 1. Introduction

Since the 1980s [1,2], with the development of Ti:Sapphire mode-locking techniques, femtosecond lasers have raised lots of concern and gradually extended to the field of processing and manufacturing. Compared with the processing based on CO_2_ and YAG lasers, femtosecond laser processing has a very small heat-affected zone (HAZ) as well as much higher accuracy [3,4,5]. In addition, compared to the excimer laser emitting the ultraviolet wavelength, femtosecond lasers can not only perform surface micromachining, but also perform welding and internal micromachining on transparent materials, which shows great advantages in the fabrication of three-dimensional photonic devices [6,7]. The mechanism of the femtosecond laser processing is different from that of long pulses because the high energy density deposition in very limited spans of time (10^−10^–10^−12^ s) can change the mode of electron absorption and movement that can effectively avoid the influence of laser linear absorption, energy transfer and diffusion on the materials [8,9]. Therefore, femtosecond laser processing has ultra-high precision and spatial resolution with strong universality. For example, the femtosecond laser can realize the machining of brittle materials (such as ceramics, transparent dielectric materials, and silicon wafer) without cracks [10,11], as well as the fine machining of flexible materials (such as biological tissue and flexible printed circuits) [12,13] and super hard materials (such as diamond and tungsten carbide) [14,15].

Mode-locking is the main approach to generate pulse duration on the order of pico- and femtosecond [16,17,18,19]. However, due to the low single pulse energy (~nJ) and low average power (~mW) [16,20], the pulse output from the oscillator cannot be used directly in processing. With limited pump power, reducing the repetition rate of the mode-locking pulses before injecting to the amplifier (typically 10^−2^–10^−6^ times from that of the seed) is the most effective way to achieve high single pulse energy amplification. An amplifier with an optical fiber as the gain medium is considered as a promising technology due to its good heat dissipation, high environmental stability, compact structure, high beam quality, as well as low cost. But the small core area of traditional optical fiber leads to the strong accumulation of nonlinear phase shift in the transmission of the femtosecond pulse, then the pulse distortion; meanwhile, the small core area is unable to withstand high-power density and is easily damaged [21,22]. Consequently, for a long time, the pulse energy of the femtosecond fiber laser has been incomparable with that of the solid-state laser, limiting its applications in industry, until the invention of photonic crystal fiber (PCF). The PCF can provide a large area of mode field and reduce the nonlinear effect while maintaining the single-mode property of the beam [23,24,25,26]. The larger numerical aperture and bandgap guidance characteristics of PCF effectively improve the coupling efficiency of pumping light and reduce the Rayleigh scattering loss. In addition, the chirped pulse amplification (CPA) technique is an effective approach for ultrashort pulses to promote high-power amplification by reducing the peak power of the seed. Current, femtosecond laser with a peak power over 10^15^ W has been demonstrated via CPA, which proves the femtosecond laser to be a powerful tool to study super intense phenomena, and also lays a foundation for the wide application of ultrafast optics in micromachining, biomedicine, and physics, etc. Typically, two grating pairs or two independent gratings are used as the stretcher and compressor in a CPA, respectively, which usually brings about increased laser cost, larger volume, and lower stability. At present, over hundred-watt power [24,25] and millijoule energy [26] of femtosecond pulses have been generated by using CPA with rod-PCF as the gain medium. To expand the applications for industrial laser processes, apart from maintaining the ultrashort pulse duration, both high repetition rate and single pulse energy are required for femtosecond lasers to achieve high throughput. However, there are few reports on this type of industrial femtosecond laser and, of which, with examples of processing [27,28].

In this paper, we report on an Yb-doped rod-type PCF based CPA system delivering 272 fs pulses and 33.2 W average power at the repetition rate of 500 kHz, corresponding to the peak power of 244 MW. Pulse stretching, compression properties, and spectral characteristics are studied. This laser system is applied to the cutting of industrial and electronic materials, showing good performance.

## 2. High-Power Femtosecond Laser System

**Figure 1:**
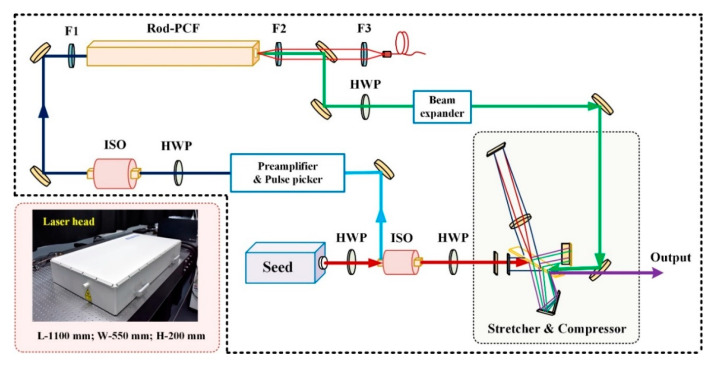
Schematic of the high-power chirped-pulse amplification (CPA) system. HWP, half-wave plate; ISO, isolator (EOT, Inc. Traverse City, MI, USA); F, focus lens. Inset is the photo of the laser head (excluding water-cooled chillers and power supply).

The schematic of the high-power CPA setup is shown in Figure 1. It consists of a femtosecond oscillator, two isolators, a double-pass preamplifier with an electro-optical pulse picker, an ytterbium-doped rod-PCF, and a dielectric-grating-based stretcher and compressor.

**Figure 2:**
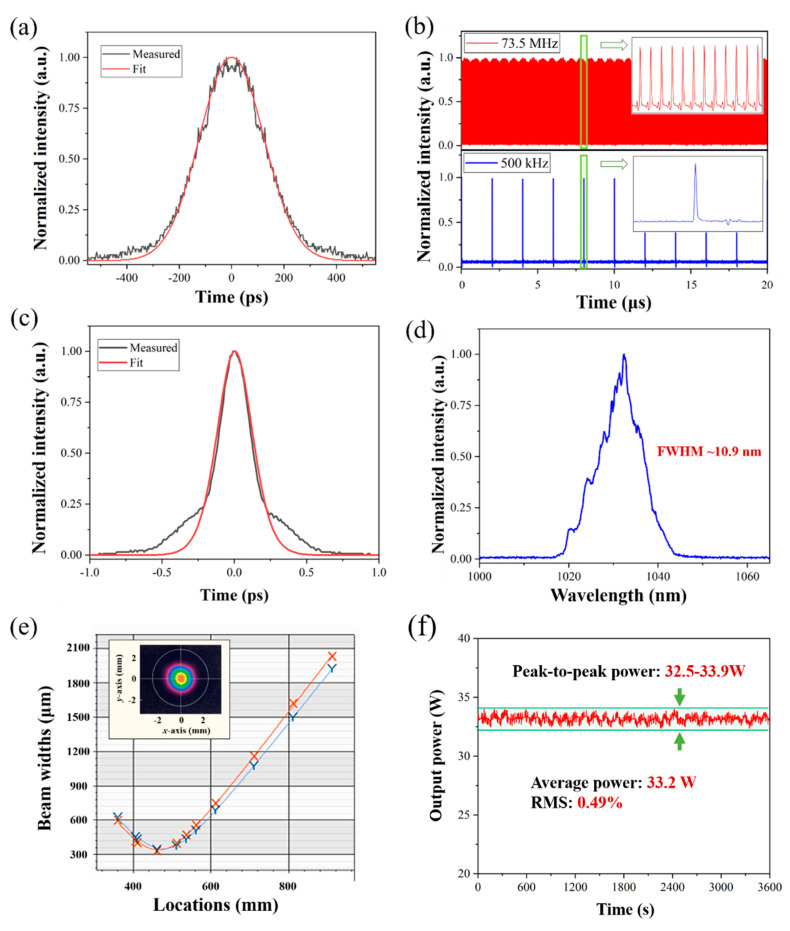
Output characteristic of the amplifier: (**a**) autocorrelation trace for the stretched pulse; (**b**) oscilloscope traces of the seed and picked pulses; (**c**) autocorrelation trace for the dechirped pulse output from the compressor; (**d**) the spectrum of the output pulse; (**e**) the beam quality and near-field profiles of the output; and (**f**) curve of the output power stability in an hour.

The diode-pumped Ytterbium femtosecond oscillator (MONTFORT Laser GmbH) runs at a 73.5 MHz repetition rate, delivering pulses 130 fs at 1045 nm center wavelength (13 nm bandwidth) and 1 W output power, corresponding to 13.6 nJ single pulse energy. After going through an isolator, the seed is injected into the stretcher and compressor (a 130 mm × 20 mm conventional gold-coated 1600 line/mm diffraction grating, LightSmyth Technologies, Inc. Eugene, OR, USA), which stretches the width of the laser pulses from 130 fs to ~200 ps (see Figure 2a). For higher pulse energy while increasing the average power, the repetition rate of the seed is reduced from 73.5 MHz to 500 kHz by the electro-optic switch driven pulse picker (EKSMA Optics). Figure 2b shows the oscilloscope trace of the seed and picked pulses. Then, the picked pulses are amplified to an average power of ~2 W via the double-pass fiber preamplifier. The main amplifier is constructed using an 80 cm long air-cladding rod-PCF (NKT photonics) with an 85 µm core and 65 µm mode-field, which is pumped by a fiber-coupled 976 nm laser diode. The stretched pulses are amplified to 42 W at the pump power of 132 W. With the same diffraction grating, the amplified pulses are dechirped to 272 fs pulse duration with a maximum output power of 33.2 W. Here, the stretching and compression limits of the pulses are mainly limited by the available size of the diffraction grating, and the power can be further increased by increasing the output power of the preamplifier. Figure 2c,d show the measured autocorrelation trace and spectral width at the highest output power, respectively. The side wings observed for the dechirped pulse (see Figure 2c) are caused by the three-order dispersion (TOD), which can be restrained by optimizing the stretcher and compressor. However, since the side wings on the spectrum have no effect on industrial processing applications, further optimization of the stretcher and compressor is not conducted. The laser output is horizontally polarized with a diameter at the exit of 3.5 mm and a divergence less than 0.04 mrad. The beam quality at the maximum output power is measured, showing Gaussian distribution of near-field with *M*^2^ = 1.1 ± 0.05 (mean value between the two orthogonal axes), as shown in Figure 2e. The small asymmetric around the focus (far-field) can be corrected by the optimization of the CPA system. To keep the optical elements from the dust and humidity, the laser is sealed by silica gel sealing ring for better environmental stability. Figure 2f illustrates the measured power fluctuation in 60 min (non-laboratory conditions at room temperature) which shows high stability with a root-mean-square (RMS) of less than 0.5%. To ensure long-term stability, the laser underwent a five-minute break after the one-hour operation; however, as far as we know, such a duty cycle can meet the requirements of most industrial applications.

## 3. Laser Processing

**Figure 3:**
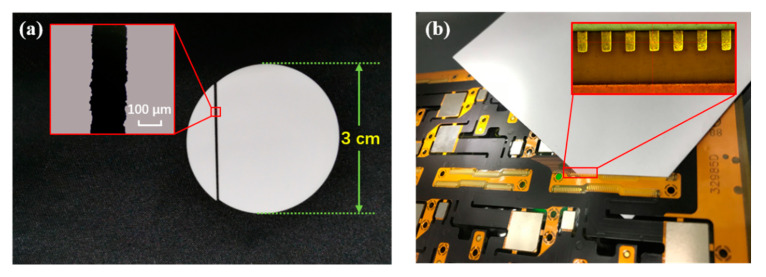
Experiment result of femtosecond laser processing of (**a**) alumina ceramic with 0.2 mm thickness, and (**b**) FPC with 0.11 mm thickness.

To test the processing performance, we applied the laser to the cutting of ceramics and the flexible printed circuit (FPC). FPC consisting of a metallic layer of traces bonded to a dielectric layer is designed as a replacement for traditional wire harnesses, however, due to the complexity of the components, realizing high precision and low damage cutting has been a research hotspot. In our cutting experiment, the beam passes a focusing lens group generating the spot diameter measured to be ~20 µm on the sample surface, and is guided by a set of x and y lenses that are moved by the galvo scanner. The traverse speed of the beam is set to be 2 mm/s, and the laser power applied is 23 W (169 MW at 500 kHz). The ceramic sample adopted in our experiment is composed of 99.6% alumina (Al_2_O_3_) with 0.2 mm thickness. Figure 3a shows the picture of the alumina ceramic after cutting and the inset is an image snapped by a microscope, which shows a smooth cut edge without any cracks on the surface. For cutting the FPC, the scan speed is set to be 2000 mm/s with the beam diameter ~20 µm at the focus, with which the 110 µm-thick FPC is cut off after 20 cutting cycles. The result is shown in Figure 3b, which indicates a good quality of cutting such as flexible materials without obvious heat effect.

## 4. Conclusions

In summary, we developed a high-power femtosecond laser system by using rod-type PCF and a single-grating-based CPA. Pulses with the duration of 272 fs and single pulse energy of 66.4 µJ are generated at 500 kHz repetition rate, corresponding to an average power of 33.2 W. Meanwhile, the laser shows high beam quality (*M*^2^ < 1.2) and high-power stability (RMS < 0.5%), even at maximum output power. The single-grating-based pulse stretching and compression structure provides high integration to the laser system, which gives rise to the overall size of the laser head only 110 × 55 × 20 cm^3^. Further power scaling to over 70 W, corresponding to the output power from the PCF amplifier ~100 W, is expected by increasing the pump power and optimizing the amplifier design (or using the coherent beam combining technique). Meanwhile, a narrower pulse-width (<100 fs) can be achieved by increasing the grating size. In addition, the high processing performance of both brittle (alumina ceramic) and flexible (FPC) materials are demonstrated with the developed laser system. Due to the small HAZ and increased energy penetration depth resulting from the high pulse intensity, the demonstrated femtosecond laser with high compactness and stability shows a great prospect in industry applications. Next, to test the industrial applicability in a complex environment, stability at significant temperature swings as well as significant vibrational stresses on the laser system will be conducted.
